# Novel PDE4 Inhibitors Derived from Chinese Medicine Forsythia

**DOI:** 10.1371/journal.pone.0115937

**Published:** 2014-12-30

**Authors:** Tiffany A. Coon, Alison C. McKelvey, Nate M. Weathington, Rahel L. Birru, Travis Lear, George D. Leikauf, Bill B. Chen

**Affiliations:** 1 Department of Medicine, Acute Lung Injury Center of Excellence, University of Pittsburgh, Pittsburgh, Pennsylvania, 15213, United States of America; 2 Vascular Medicine Institute, University of Pittsburgh, Pittsburgh, Pennsylvania, 15213, United States of America; 3 Department of Environmental and Occupational Health, University of Pittsburgh, Pittsburgh, Pennsylvania, 15213, United States of America; University of North Dakota, United States of America

## Abstract

Cyclic adenosine monophosphate (cAMP) is a crucial intracellular second messenger molecule that converts extracellular molecules to intracellular signal transduction pathways generating cell- and stimulus-specific effects. Importantly, specific phosphodiesterase (PDE) subtypes control the amplitude and duration of cAMP-induced physiological processes and are therefore a prominent pharmacological target currently used in a variety of fields. Here we tested the extracts from traditional Chinese medicine, *Forsythia suspense seeds*, which have been used for more than 2000 years to relieve respiratory symptoms. Using structural-functional analysis we found its major lignin, Forsynthin, acted as an immunosuppressant by inhibiting PDE4 in inflammatory and immune cell. Moreover, several novel, selective small molecule derivatives of Forsythin were tested *in* *vitro* and in murine models of viral and bacterial pneumonia, sepsis and cytokine-driven systemic inflammation. Thus, pharmacological targeting of PDE4 may be a promising strategy for immune-related disorders characterized by amplified host inflammatory response.

## Introduction

Phosphodiesterase (PDE) is an enzyme that catalyzes the hydrolysis of a phosphodiester bond, most notably those of the second messenger cascade molecules cyclic adenosine monophosphate (cAMP) and cyclic guanosine monophosphate (cGMP). cAMP and cGMP bind to the regulatory units of protein kinase A(PKA) allowing for phosphorylation thus transducing signal cascades in the cell [1]–[3]. Consequently, by severing these cA/GMP-dependent pathways, PDE has an enormous clinical significance monitoring cAMP and cGMP levels in cells. The PDE superfamily comprises 11 subfamilies, PDE1-11, respectively, each utilizing different substrate specificities. For example, PDE4, 7, and 8 strictly hydrolyze cAMP; PDE5, 6, and 9, on the other hand, only hydrolyze cGMP while the other family members (PDE1, 2, 3, 10, 11) can target both cAMP and cGMP for hydrolysation [4]. For many years, PDE enzymes have remained an interest within the pharmaceutical industry, as inhibition of PDE can increase the levels of cAMP or cGMP thus enhancing or prolonging their natural physiological effects [5].

Currently, various small molecular compounds have been discovered to inhibit PDEs. For example, caffeine, aminophylline, and IBMX are nonselective PDE inhibitors [6] that increase intracellular cAMP, thereby activating PKA, thus inhibiting tumor necrosis factor (TNF) and other inflammatory cytokines, and reducing inflammation [7]. There are also selective PDE inhibitors. Specifically, PDE3 inhibitors such as inamrinone and milrinone, are used for short-term treatment of congestive heart failure [8], and Cilostazol used in the treatment of intermittent claudication [9]. The PDE5 inhibitors Sildenafil, tadalafil and vardenafil boost cGMP levels in penile tissue [10]–[12] and are used primarily for erectile dysfunction, as well as having secondary indication in treatment of pulmonary hypertension [13], [14]. Recently_,_ an opium alkaloid, Papaverine, was shown to inhibit PDE10 [15]. Among all of the PDE isoforms, PDE4 is the major cAMP-degrading enzyme found in inflammatory and immune cells. Selectively inhibiting PDE4 prevents the release of cytokines and other inflammatory factors and hinders the production of reactive oxygen species [16], [17].

As the leading causes of infectious deaths in the US, sepsis and pneumonia are pathognomonically linked to a burst in cytokine release, i.e. cytokine storm, from pro-inflammatory cells including macrophages, lymphocytes, and polymorphonuclear leukocytes [18] [19]. Cytokine storm occurs in response to infection with virulent pathogens, host cell injury, or irritants that activate a multitude of receptors on immune effector cells. Under some conditions it is exaggerated (hypercytokinemia) and results in a fatal immune reaction with constant activation of immune effector cells that produce sustained or supraphysiologic levels of tumor necrosis factor (TNF), interleukin 1 (IL1), and interleukin-6 (IL6) that results in severe tissue injury and often death. Thus, selectively inhibiting PDE4 may ameliorate cytokine storm and prevent tissue injury.

The earliest Pharmacopoeia in the world, the “Tang Ben Cao”, completed in 659 AD is the first known documentation of the *Forsythia suspensa* plant being used to treat fever, flu-like, and inflammatory symptoms. Since then, it has been habitually and widely used in Asia as one of the major traditional medicines. Nowadays, the extract of *Forsythia suspensa* seeds is used to treat numerous inflammatory diseases including but not limited to erysipelas, inflammation, pharyngitis, pyrexia, tonsillitis, and ulcers [20], [21]. Moreover, it has been shown in studies that the crude extract displays potential antibacterial, antiviral, choleretic and antipyretic effects [22]. Medicinal chemistry studies on Forsythia seeds have revealed phenolic compounds including lignans and flavonols which are suspected to be responsible for the various biological activities of the herb [22], [23]. In this study, we sought to better understand *Forsythia* lignan Forsythin structure-function relationships and it was used to design and test a new class of selective PDE4 inhibitors. These selective PDE4 inhibitors exert potent anti-inflammatory activity in several murine models of inflammation.

## Results

### Forsythin is an *in* *silico* inhibitor of PDE4

**Figure 1:**
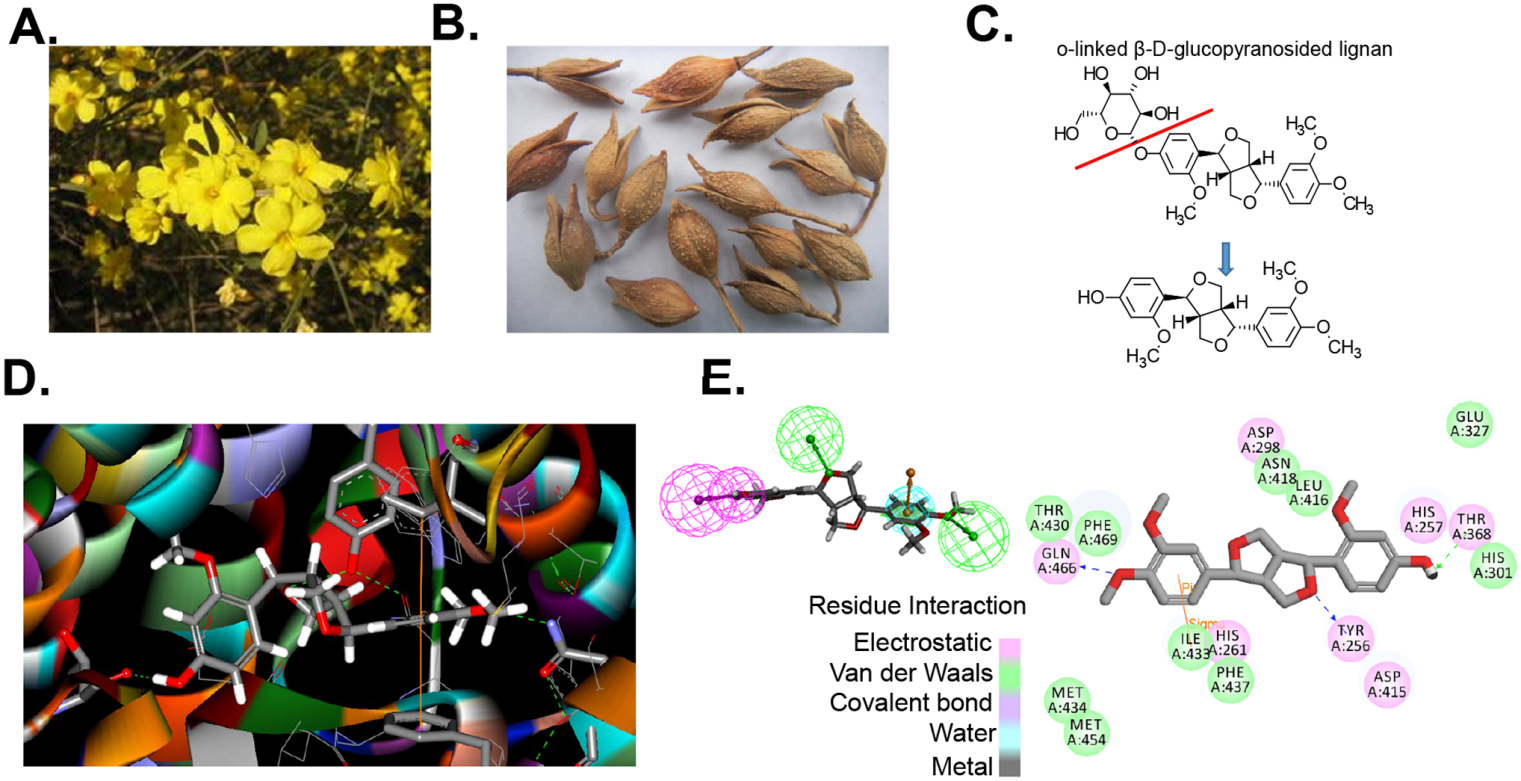
Forsythin is an inhibitor of PDE4. **A–C.** The chemical structure of Forsythin extracted from *Forsythia suspensa* seeds. Forsythin is an o-linked β-D-glucopyranosylated lignin that can be hydrolyzed (Red line). **D.** Predicted docking site of Forsythin with PDE4. **E.** Predicted residue electrostatic and van der Waals interactions between PDE4 amino acids and Forsythin. Using ZINCPharmer [24], a pharmacophore model was generated (upper left) and used to screen the lead compounds from an 18.3 million purchasable compound library.

**Figure 2:**
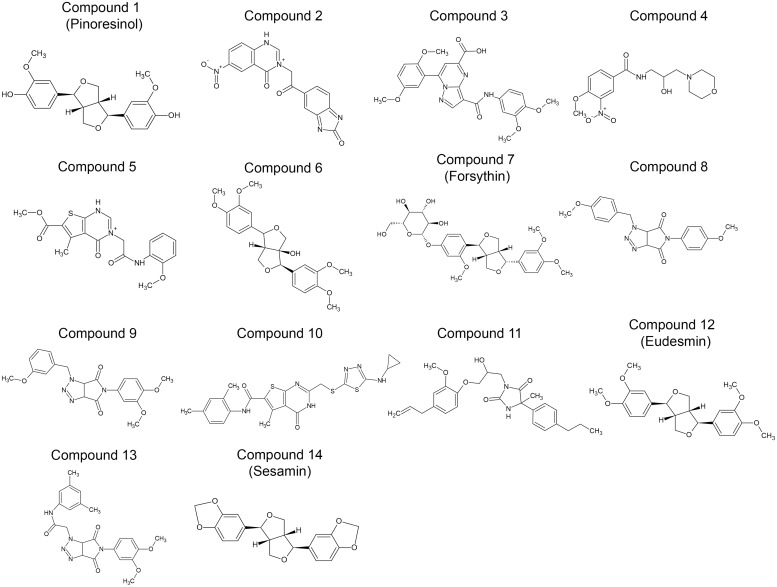
Molecular structures of selected compounds. Over 100 high value lead compounds were generated through ZINCPharmer. These compounds were then refined by a computer based docking analysis using LIBDOCK (Discovery Studio 3.5). Among the top scoring compounds, 14 compounds were selected for further testing using an *in* *vitro* phosphodiesterase activity assay.

Forsythin is one of the major bioactive compounds extracted from *Forsythia suspensa* seeds (Fig. 1A–B) (27). An o-linked β-D-glucopyranosylated lignan, Forsythin readily can be hydrolyzed to remove a glucose moiety (Fig. 1C). Using the structure of the predicted metabolite, we assessed the virtual docking with PDE4D (based on PyMol PDB 1 mkd.phg) using LibDock (Discovery Studio, Accelrys Software Inc./BIOVIA, San Diego, CA) (Fig. 1D,E). Using ZINCPharmer [24], we then generated a pharmacophore model (Fig. 1E top left) that enabled the identification of over 100 high value targets from a 18.3 million purchasable compound library. We refined this search by performing a computer based docking analysis of the target compounds using LibDock (Discovery Studio 3.5) and selected 14 compounds (Fig. 2) for further testing using an *in* *vitro* phosphodiesterease activity assay.

### Tested compounds exhibit high potency and selectivity towards PDE4

**Figure 3:**
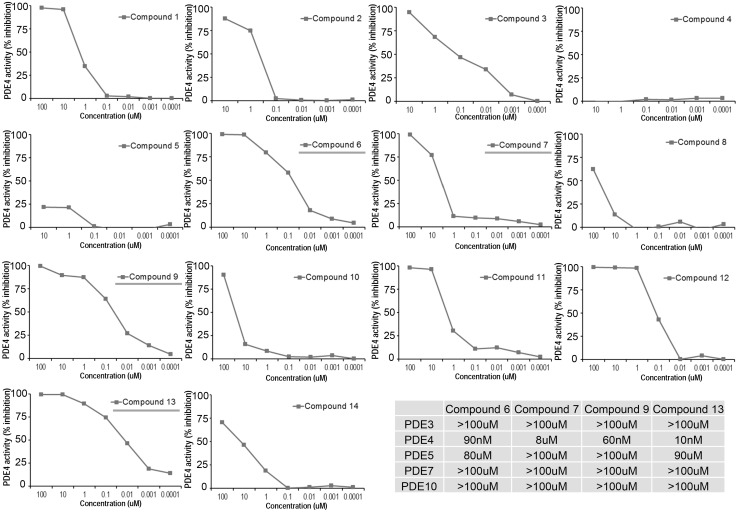
Tested compounds exhibit high potency and selectivity towards PDE4. For the PDE activity assay, all test compounds were diluted in DMSO with final concentrations in each assay of 100, 10, 1, 0.1, 0.01, 0.001, 0.0001 µM. For the PDE4 activity assay, 10 mU of purified PDE4 (Millipore) was used per reaction. Compounds 6, 7, 9 and 13 (blue underline) were further tested in PDE3, 5, 7, 10 activity assays in which 25 mU of purified enzyme was used per reaction. Summary of compound IC_50_ in lower right corner.

Compounds 1–14 (Fig. 2) were tested *in* *vitro* using PDE-glo phosphodiesterase assay (Promega, Madison, WI). Briefly, all the compounds were diluted in DMSO, and the final concentrations in each assay were 100, 10, 1, 0.1, 0.01, 0.001, 0.0001 µM. For the PDE4 activity assay, 10 mU of purified PDE4D (Millipore) was used per reaction. Compound 7, which is Forsythin, exhibited [IC_50_] PDE4 = 8 µM. Other custom designed compounds such as 6, 9 and 13 exhibited much greater potency with an [IC_50_] towards PDE4 ranging from 10–90 nM. Impressively, all of the test compounds exhibited selectively toward PDE4, in as much as the [IC_50_]>80 µM with PDE3, 5, 7, and 10 (Fig. 3).

### Tested compounds reduce TNF secretion in LPS stimulated mouse RAW264.7 and human PBMCs

**Figure 4:**
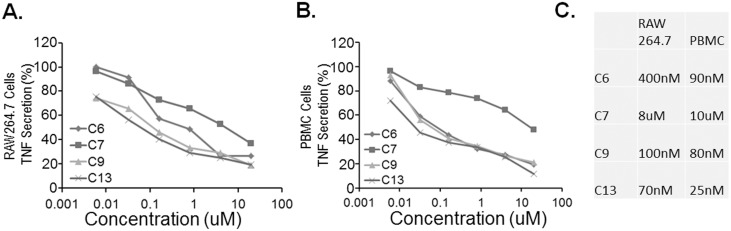
Tested compounds reduce TNF secretion in LPS stimulated mouse RAW264.7 and human PBMCs. **A.** 5×10^5^ RAW264.7 cells were seeded in 96 wells for 18 h. Cells were primed with compounds at different concentration for 3 h before treated with LPS (1 ng/ml) for additional 8 h. TNF cytokine releases were monitored by ELISA. **B.** PBMC (0.2 ml at 1×10^5^/ml) were primed with compounds at different concentration for 3 h before treated with LPS (1 ng/ml) for additional 8 h. TNF cytokine releases were monitored by ELISA. % of TNF secretion were calculated and graphed. **C.** Summary of compound IC_50_. The data represent *n* = 3–6 experiments.

Compound 6, 7, 9 and 13 were also tested in LPS stimulated RAW264.7 (Fig. 4A) and PBMC (Fig. 4B) cells to evaluate their ability to suppress TNF secretion. Compound 7, which is Forsythin, exhibited [IC_50_] TNF = 8–10 µM in RAW264.7 and PBMC cells, which is comparable to its [IC_50_] PDE4. Other custom designed compounds such as 6, 9 and 13 exhibited much greater potency with an [IC_50_] towards TNF ranging from 25–400 nM. Specifically, compound 13 exhibited greatest potency towards PDE4 with [IC50] = 10 nM (Fig. 3), it also exhibited comparable potency towards [IC_50_] TNF ranging from 25–75 nM (Fig. 4C).

### PDE4 inhibitors ameliorate LPS-induced lung injury

**Figure 5:**
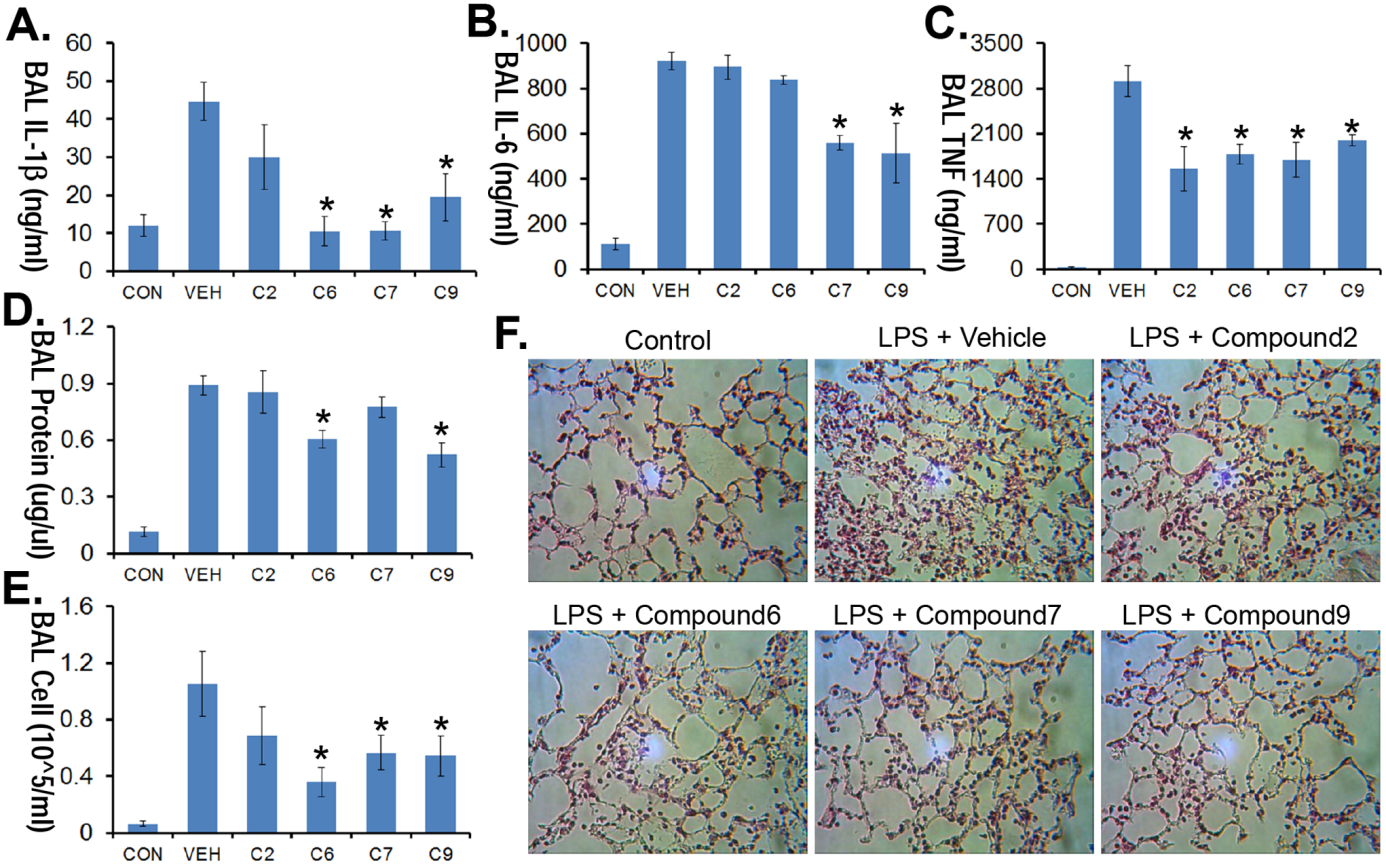
PDE4 inhibitors ameliorate LPS induced lung injury. C57/BL6 mice were challenged with LPS *(E.coli*, 3 mg/kg, i.t.) followed by i.p. administration of vehicle, 1 mg/kg of compound 2, 6, 7 or 9. Mice were then euthanized 18 h later using pentobarbital, and lungs were lavaged with saline, harvested, and then homogenized. Lavage cytokine secretion (**A–C**), protein concentrations (**D**) and cell counts (**E**) were measured. **F.** H&E staining was performed on lung samples. Original magnification, ×20. The data represent *n* = 4–6 mice/group, *P<0.05 versus vehicle.

To assess any *in* *vivo* anti-inflammatory activity, four compounds [C2, C6, C7 and C9, 1 mg/kg, intraperitoneal (i.p.) injection] were administered in a LPS-induced pneumonia model. Briefly, compounds were administered to mice at various doses through an i.p. injection 10 min after mice were given LPS (i.t. 3 mg/kg). Mice were then euthanized 16 h later using pentobarbital; BAL was collected and assayed for IL1, IL6 and TNF cytokine levels. These compounds varied in their ability to ameliorate lavage cytokines, protein concentration, and total cell counts from LPS-treated mice (Fig. 5A–E). In addition, these inhibitors diminished histological evidence of lung injury (Fig. 5F).

### PDE4 inhibitors lessen cytokine storm induced by LPS septic shock

**Figure 6:**
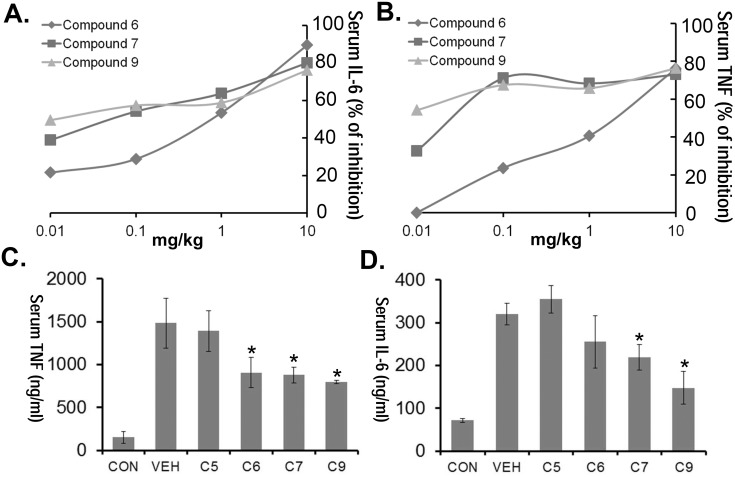
PDE4 inhibitors lessen cytokine storm induced by LPS septic shock. C57/BL6 mice were administered i.p. nothing (CON), vehicle, 10 ug/kg, 100 ug/kg, 1 mg/kg or 10 mg/kg of compounds 6, 7 or 9. Mice were given LPS (*E. coli,* 100 µg) 10 min later through an i.p. injection. 2 h later mice were euthanized using pentobarbital and blood was collected for IL-6 and TNF measurements. Shown in panel **A–B** is the % inhibition of cytokine levels as a function of drug dose. The data represent *n* = 3 mice/group at each dose. C57/BL6 mice were also pretreated with compound 5, 6, 7 or 9 at 1 mg/kg. Mice were given LPS (*E. coli,* 100 µg) 18 h later through an i.p. injection. 2 h later the mice were euthanized using pentobarbital and blood was collected for IL-6 and TNF measurements (**C–D**). The data represent *n* = 4–6 mice/group, *P<0.05 versus vehicle.

As a widely used model of sepsis [25], selective PDE4 inhibitors (C6, 7 and 9) were administered to mice at various doses through an i.p. injection, and 10 min later mice were given LPS (*E. coli,* 100 ug i.p.). Mice were euthanized 2 h later using pentobarbital where blood was collected and assayed for IL6 and TNF cytokine levels. All inhibitors exhibited high potency *in* *vivo* (inhibitory dose [ID_50_] TNF = 0.01∼1 mg/kg, ID_50_ IL-6 = 0.1∼1 mg/kg,) (Fig. 6A–B). In a separate experiment, a brief pharmacokinetic study was performed where PDE4 inhibitors (1 mg/kg, i.p.) were given to mice 18 h before LPS administration (*E. coli,* 100 ug i.p.). Compound 9 demonstrated excellent efficacy reducing both serum TNF and IL-6 cytokines even after 18 h lead time (Fig. 6C–D).

### PDE4 inhibitors reduce H1N1 influenza-induced lung injury

**Figure 7:**
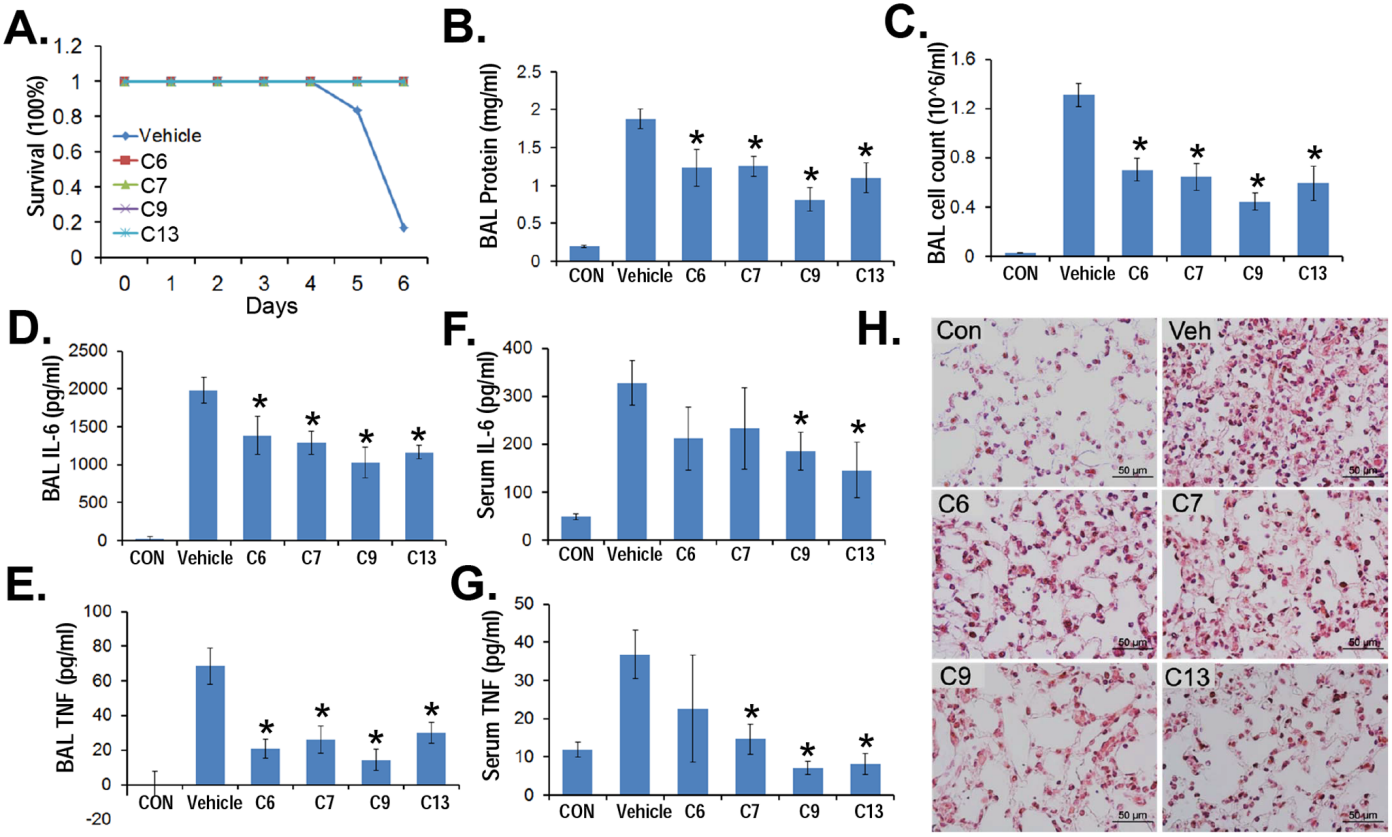
PDE4 inhibitors reduce H1N1 influenza-induced lung injury. C57/BL6 mice were challenged with H1N1 (10^5^ pfu/mouse, i.t.) for up to 6 days. For compound treatment, a stock solution (5 mg/ml) was added to the drinking water (containing 2% sucrose) to a final concentration of 30 µg/ml. **A.** Survival studies of mice administered i.t. with H1N1 (10^5^ pfu/mouse, *n* = 6 mice/group). Mice were then euthanized using pentobarbital and lungs were lavaged with saline, harvested, and then homogenized. Cell counts and lavage protein were measured (**B, C**). Lavage cytokine secretions were measured (**D, E**). Serum samples were also collected and cytokine levels were measured (**F, G**). **H.** H&E staining was performed on lung samples. Original magnification, ×60. The data represent *n* = 4–6 mice/group, *P<0.05 versus vehicle.

To further test these compounds, mice were challenged with H1N1 (105 pfu/mose i.t.) without (vehicle control) or with C6, C7, C9, or C13 treatment (30 µg/ml added to drinking water containing 2% sucrose: estimated dose = 5 mg/kg/d). All four PDE4 inhibitors significantly improved the survival of mice infected with H1N1 compared with mice receiving diluent (Fig. 7A). The PDE inhibitors also decreased lavage cell counts, protein concentrations, and lavage cytokine IL-6 and TNF (Fig. 7B–E). Impressively, PDE4 inhibitors also significantly reduced serum cytokines induced from the H1N1-induced systematic inflammation (Fig. 7F,G). The tested compounds were also able to lessen signs of tissue injury (Fig. 7H). These results suggest that the selective PDE4 inhibitors suppress inflammation and preserve lung homeostasis after pulmonary H1N1 infection.

## Discussion

It has been known for years that increased cAMP from PDE4 inhibition or gene knockout were able to markedly decrease inflammatory responses in immune cells [26]. cAMP controls inflammation through several distinct pathways, including cyclic nucleotide-gated ion channels, cAMP-activated protein kinases (PKA), or exchange proteins directly activated by cAMP (Epac) [27]. Specifically, cAMP actives PKA, negatively regulates NF-KB pathway, thus suppressing the release of proinflammatory mediators (eg, TNF-α, interleukin-17, Interferon-γ) [28]. Moreover, cAMP actives transcriptions of anti-inflammatory gene such as interleukin-10 through transcription factor CREB (cAMP response element-binding protein) [29]. Thus, pharmacological targeting of PDE4 may be a promising strategy for anti-inflammatory therapy.

Here we developed a novel class of selective PDE4 inhibitors that exert robust anti-inflammatory activity by impairing cytokine release in three preclinical models. The structural basis for our drug design emerged from Forsythin, the one of major compound extracted from *Forsythia suspensa* seeds. Among the 14 compounds tested in this study, synthetic compounds 6, 9 and 13 showed the potent IC_50_ (10–90 nM) toward PDE4. Impressively, these compounds also exhibit high selectivity toward PDE4, with >80 µM IC_50_ towards other PDE enzymes (Fig. 3). Compounds 6, 9 and 13 also showed potent IC_50_ (20–450 nM) toward TNF inhibition in LPS stimulated RAW274.7 and PBMC cells. Many natural compounds, such as the flavonoid derivatives Hesperetin and prunetin, were also isolated from traditional Chinese medicines are selective inhibitors of PDE4 with an IC_50_ ranging from 10–100 µM [30]. However, little is known about the structure–activity relationship between such lignans or their inhibitory effect on proteins in the inflammatory pathway. In this study, we determined that o-linked β-D-glucopyranosided lignan Forsythin is a weak but selective inhibitor of PDE4. Structurally speaking, compound 6 and Forsythin are highly similar except that Forsythin is an o-linked β-D-glucopyranoside. *In vitro*, Forsythin is >80 times weaker in activity toward PDE4 compared with compound 6. However, our *in* *vivo* study suggested that Forsythin and compound 6 exhibit similar activities in several murine models (Fig. 5A, 6C, 7B). This suggests that Forsythin may act as a prodrug that its glucopyranoside group will be potentially removed *in* *vivo* by β-glucosidase, and release the active lignin; which is a common of metabolic reaction in plant lignans and flavonols [31], [32].

Pandemic H5N1 *Influenza* virus and *SARS* virus are known to cause dangerous cytokine storm resulting in severe damage to alveoli and lung tissue and thus extremely high mortality rates [33], [34]. Today, viral pneumonia affects more than 100 million children and 100 million adults per year yet there are no effective treatments only supportive care [35]. Our H1N1 murine model recapitulates well with the deadly human viral pneumonia, as H5N1 *Influenza* infection is largely driven by an exuberant host response and amplification of cytokines and chemokines that contribute to influenza-induced morbidity and mortality [25], [36], [37]. Our pulmonary viral infection model was complemented with a murine sepsis model to provide assessment of PDE4 inhibitors on systemic inflammation by the endotoxin LPS [37], [38]. Importantly, in each of these models of inflammation, our PDE4 inhibitors (Compound 6, 7, 9, 13) were observed to lessen severity of tissue injury using varying modes of application (parenteral, oral), and none of the animals treated with the agents exhibited overt signs of distress. The dosing of these compounds also appeared to be excellent *in* *vivo*, with <1 mg/kg efficacious dose in both LPS pneumonia and sepsis model. Specifically, these compounds also appeared to have long half-life *in* *vivo*. As shown in Fig. 6C–D, the septic shock model was carried out 18 h after the initial treatment (1 mg/kg, i.p.), yet we still observed a 40–60% decrease in serum cytokines.

Overall, our pre-clinical studies demonstrate the biological efficacy of selective PDE4 inhibitors in murine models where both infectious and irritant factors trigger cytokine release. These selective PDE4 inhibitors could potentially have broad applications in acute and chronic inflammatory diseases. In fact the selective PDE4 inhibitor, Roflumilast, recently became the first FDA approved PDE4 inhibitor on the market [39], [40]. Roflumilast is a potent anti-inflammatory drug, particularly in pulmonary diseases such as asthma, COPD, and rhinitis. Thus, PDE4 is a potentially important drug target for the treatment of inflammatory diseases. However, further research and development clearly is needed to carefully ascertain the safety profile, distribution, elimination, and metabolism of these new chemical entities in larger models of inflammation. Successful results from these pharmacokinetic studies will set the stage for transition to clinical testing in subjects with acute and chronic immune-related illness.

## Methods

### Materials

RAW 264.7 cells were from ATCC. PBMCs were from Sanguine Life Sciences. PDE4 activity assay kit was from Promega. IL1β, TNFα, IL6 mouse ELISA kit, human TNFα were from R&D systems. LPS (E.coli) were from Sigma. Forsythin was from Stanford Chemicals; all other synthetic compounds were purchased from ChemDiv, Inc. and Vitas-M Laboratory, Ltd. All compounds are >98% pure by HPLC.

### Animal studies

All animal experiments were approved by the University of Pittsburgh Institutional Animal Care and Use Committee (IACUC) under protocol (14023127). Mice were housed at University of Pittsburgh Animal Care Facility and maintained according to all federal and institutional animal care guidelines.

#### Method of sacrifice

All mice were euthanized using i.p. injection of 100 mg/kg pentobarbital approved by University of Pittsburgh Institutional Animal Care and Use Committee (IACUC) under protocol (14023127).

### Sepsis model

Male C57LB/6 mice (purchased from Jackson Laboratories) were acclimated at the University of Pittsburgh Animal Care Facility and maintained according to all federal and institutional animal care guidelines and under a University of Pittsburgh Institutional Animal Care and Use Committee (IACUC)-approved protocol (14023127). Male C57LB/6 Mice were deeply anesthetized with ketamine (80 to 100 mg/kg of body weight, intraperitoneally [i.p.]) and xylazine (10 mg/kg, i.p.). Compounds were diluted in PBS and various amounts (0.01, 0.1, 1 and 10 mg/kg) of compounds were administered to mice though an i.p. injection. 10 min later, mice were given 100 µg of LPS (*E. coli*) through an i.p. injection. 2 h later, mice were euthanized. Plasma was collected and processed for cytokine assays.

### LPS Pneumonia modes

Male C57LB/6 mice (purchased from Jackson Laboratories) were acclimated at the University of Pittsburgh Animal Care Facility and maintained according to all federal and institutional animal care guidelines and under a University of Pittsburgh Institutional Animal Care and Use Committee (IACUC)-approved protocol (14023127). Male C57LB/6 Mice were deeply anesthetized with ketamine (80 to 100 mg/kg of body weight, intraperitoneally [i.p.]) and xylazine (10 mg/kg, i.p.), and then the larynx was well visualized under a fiber optic light source before endotracheal intubation with a 3/400 24-gauge plastic catheter. Compounds were diluted in PBS and administered to mice at 1 mg/kg through an i.p. injection 10 min after mice were given with LPS (i.t. 3 mg/kg). Mice were then euthanized 18 h later; Lavage fluids were collected from mice to measure protein and cytokine concentration. Lungs were isolated from mice for H&E staining.

### H1N1 Pneumonia model

Male C57LB/6 mice (purchased from Jackson Laboratories) were acclimated at the University of Pittsburgh Animal Care Facility and maintained according to all federal and institutional animal care guidelines and under a University of Pittsburgh Institutional Animal Care and Use Committee (IACUC)-approved protocol (14023127). Male C57LB/6 Mice were deeply anesthetized with ketamine (80 to 100 mg/kg of body weight, intraperitoneally [i.p.]) and xylazine (10 mg/kg, i.p.), and then the larynx was well visualized under a fiber optic light source before endotracheal intubation with a 3/400 24-gauge plastic catheter. 50 ul of H1N1 (A/PR/8/34, 10^5^ pfu/mouse) was instilled i.t. and mice monitored for up to 6 d. For compound treatment, a stock solution (5 mg/ml) was added to the drinking water (containing 2% sucrose) to a final concentration of 30 µg/ml. For survival studies, mice were carefully monitored and weighted over time; mice that lost >20% weight and moribund, preterminal mice were immediately euthanized and recorded as deceased. Lavage fluids were collected from mice to measure protein and cytokine concentration. Lungs were isolated from mice for H&E staining.

### In silico docking study

Docking study was carried using Libdock program within Discovery Studio 3.5. PDE4D crystal structural 1MKD.pdb was used in this docking study. Protein cavity and potential drug binding site were first determined (X: 44.976, Y: 7.389, Z: 124.713, radius 10.8). Libdock program parameters were: Number of Hotspots: 100, Docking tolerance: 0.25, Docking Preference: High quality, Conformation method: Fast, Minimization Algorithm: Do not minimize, Parallel Processing: True. After the docking study, the top score-ranking molecules were selected and evaluated further.

### PDE activity assay

A PDE activity assay was carried out using the PDE-Glo Phosphodiesterase Assay per manufacturer’s instruction (Promega). Briefly, 5 ul of 1X PDE-Glo Reaction buffer containing 10 mU of purified human recombinant PDE4A4 (Millipore) was added to 96 well plate. All test compounds were dissolved in DMSO and a serial dilution (110) of the inhibitors were performed using 1X PDE-Glo Reaction buffer. 7.5 ul of test compounds were added to the PDE enzyme with the final concentrations in each assay of 100, 10, 1, 0.1, 0.01, 0.001, 0.0001 µM, and incubated at room temperature for 10 min. To initiate the PDE reaction, 12.5 ul of 2 µM cAMP was added to to each reaction, mixed well and incubate at room temperature for 10 min. The reactions were terminated using 12.5 ul of PDE-Glo Termination Buffer. 12.5 ul of PDE-Glo Detection Solution containing PDE-Glo Detection Buffer and Protein Kinase A was added to each reaction. The reactions were carried at room temperature for 20 min. 50 ul of Kinase-Glo Reagent was then added to each reaction, mixed well, and incubate for 10 minutes at room temperature. The chemical luminescence signals were measured, quantified and graphed. Compounds 6, 7, 9 and 13 were further tested similarly using 25 mU purified PDE3, 5, 7, 10 (Millipore) enzyme.

### LPS induced cytokine release in RAW264.7 and PBMC cells

5×10^5^ RAW264.7 cells were seeded in 96 wells for 18 h. Cells were primed with compounds at different concentration for 3 h before treated with LPS (1 ng/ml) for additional 8 h. TNF cytokine releases were monitored by ELISA. PBMC (0.2 ml at 1×10^5^/ml) were primed with compounds at different concentration for 3 h before treated with LPS (1 ng/ml) for additional 8 h. TNF cytokine releases were monitored by ELISA. % of TNF secretion were calculated and graphed.

### Statistical Analysis

Statistical comparisons were performed with the Prism program, version 4.03 (GraphPad Software, Inc., San Diego, CA) using an ANOVA 1 or an unpaired 2 t-test with p<0.05 indicative of significance.
